# Comparison of single and double centrifugation methods for preparation of Platelet-Rich Plasma (PRP)

**DOI:** 10.12669/pjms.39.3.7264

**Published:** 2023

**Authors:** Nazish Saqlain, Naghmana Mazher, Tooba Fateen, Asma Siddique

**Affiliations:** 1 Nazish Saqlain, FCPS Hematology, Associate Professor of Pathology. The Children’s Hospital and UCHS, Lahore, Pakistan; 2 Naghmana Mazher, M.Phil Hematology, Associate Professor of Pathology. Fatima Jinnah Medical University, Lahore, Pakistan; 3 Tooba Fateen, FCPS Hematology, Associate Professor of Pathology. Allama Iqbal Medical College, Lahore, Pakistan; 4 Asma Siddique, B.Sc Medical Laboratory Technologist. The Children’s Hospital and UCHS, Lahore, Pakistan

**Keywords:** Platelet-rich plasma, Platelet concentration, Centrifugation, Blood

## Abstract

**Objective:**

To compare the platelet count, platelet concentration/yield, residual Red blood cells (RBCs) and White blood cells (WBCs) counts in platelet-rich plasma (PRP) samples prepared from the single- and the double-centrifugation protocols.

**Methods:**

It was a Cross-Sectional study, conducted at the Department of Hematology & Transfusion Medicine, The Children’s Hospital and UCHS, Lahore from October 2021 to January 2022 including 50 voluntary, healthy individuals of age 20-45 years of both genders, after taking informed consent. Complete blood count analysis of all participants was done initially by drawing 3ml blood in EDTA vial. From all the participants, 20 ml venous blood sample was taken in syringes containing tri-sodium citrate and then shifted to harvest tubes. Group-I comprised of PRP samples prepared by single- centrifugation method. While Group-II samples were prepared by Double-centrifugation method consisting of soft and hard spin. The platelet, RBC and WBC counts in prepared PRP samples were determined by using automated SYSMEX XP-100 hematology analyzer. Platelet yield or Platelet concentration (%) was calculated for samples using formula. The data analysis was done using SPSS version 23.

**Results:**

The mean PRP platelet count in Group-I was 594.6±157.4×10^3^/μl whereas in Group-II was 923.06 ± 127.58×10^3^/μl. In Group-I, the mean platelet concentration/yield in PRP was 175.75 ± 55.08% while in Group-II, it was 276.78 ± 112.7%. Significant difference was observed between the platelet counts and platelet concentration/yields from the PRP samples of two Group-s (p < 0.01). Significant difference between the WBCs count was also observed (p < 0.01) with higher WBCs in Group- I PRP. Residual RBCs were almost same among two Group-s.

**Conclusions:**

The double centrifugation protocol resulted in higher platelet quantity and yield with less contamination by red and white blood cells than did the single centrifugation protocol for PRP preparation. So, double centrifugation method is beneficial in preparation of autologous as well as allogenic PRP.

## INTRODUCTION

The Platelet Rich Plasma (PRP) is derived from venous blood and as the name indicates it has high quantity of platelets, ideally in the range of 200 to 1000x10^3^ /mL.^1^ The platelets present in the PRP, can be used in enhancing the concentration of growth factors. PRP is a promising treatment modality with proven safety for certain musculoskeletal disorders, maxillofacial surgeries, and other implant and pre-implant procedures.^2^ Clinical effectiveness has been documented in knee osteoarthritis as well.^3^

The therapeutic effects of PRP are due to the high levels of growth factors secreted by platelets such as platelet-derived growth factor AB (PDGF-AB), vascular endothelial growth factor (VEGF), epidermal growth factor (EGF) transforming growth factor beta-1 (TGF-β1) and many others along with a number of synergistically acting cytokines. These factors play a crucial role in the beginning phase of wound recovery and promote tissue repair by cell proliferation, angiogenesis, and cell migration. In addition, the PRP is effective in rapid bone regeneration and faster hemostasis.^4^

The efficacy of PRP is highly affected by its method of preparation. The centrifugal conditions for the preparation of pure platelet-rich plasma (P-PRP) affect the cellular composition of PRP obtained; however, the most favorable centrifugal conditions to prepare P-PRP are still under discussion and a single standardized method is not mentioned in literature.^5^,^6^ In this study, we have tried to establish an optimal and simple method of PRP preparation in resource-constrained settings by comparing single and double centrifugation protocols to obtain an ideal platelet count and yield with minimal alteration to platelets physiology.

The previous studies have focused on mainly platelet count but we have included comparison of Red and White blood cells (RBCs & WBCs) in prepared PRP also, keeping in mind the use of allogenic PRP in cases where autologous product would not have been possible attributing to patient or blood products storage conditions. The objective of the study was to compare the platelet count and concentration/yield, residual RBCs and WBCs in platelet-rich plasma (PRP) samples prepared from the single- and the double-centrifugation protocol.

## METHODS

It was a Cross-Sectional study, conducted at the Department of Hematology & Transfusion Medicine, The Children’s Hospital and UCHS, Lahore from October 2021 to January 2022 after receiving approval from the hospital Institutional Review Board (manuscript no: 1434/SAHS dated 06/12/2021). In this study, 50 voluntary individuals of age 20-45 years of both genders were included after taking informed consent. Medical history was taken on a pre-designed proforma to rule out any acute or chronic illness, based on WHO (World Health Organization) blood donor selection criteria.^7^

### Single- Centrifugation Protocol (Group- I):

Ten mL of venous whole blood sample was collected into syringe containing tri-sodium citrate (40mg/ml) as an anticoagulant and then transferred to clean harvest tube. The tubes were covered and placed in a table-top lab centrifuge (ELMI Centrifuge CM-MT) and centrifuged at 3500 rpm for 10 minutes. The top clear plasma (1.5-2ml) was removed leaving the bottom contents. The remaining plasma layer plus 0.5ml of the red layer was taken and mixed. The method was adopted from the study done by Harrison T.E et al.^8^ The measures were then performed and results noted (Group-I).

### Double- Centrifugation Protocol (Group-II):

Another 10 ml whole blood sample was collected by using the protocol as mentioned above. The tube was centrifuged at 1000 rpm for five minutes to separate red blood cells, buffy coat (containing white blood cells) and plasma from bottom to top (soft spin). The plasma and superficial buffy coat part were transferred into other tube and centrifuged further at 800 rpm for 10 minutes (hard spin) to obtain a platelet pellet at the bottom of the tube and platelet-poor plasma (PPP) in the upper part. Two-third of this plasma was removed and the platelet pellet was re-suspended in one-third plasma, which was then tested.^9^ By this method 2-2.5ml of PRP obtained.

### Measures and Analysis:

Complete blood count analysis (CBC) was done for all venous whole blood samples (3ml) taken in EDTA vial, before inclusion in the study to ensure platelets within normal range (150-450 ×10^3^/μl) and the count was recorded. The platelet count and residual RBCs and WBCs count of PRP samples from both Group-s (I & II) were checked and noted. All measures were done using automated SYSMEX (XP-100) hematology analyzer in duplicate and average of two result values taken into analysis. Platelet yield or platelet concentration (%) measurement was derived from the study by Tamimi et al and Nagata MJ et al.^10^,^11^ It was calculated by using the formula as given:

Platelet Yield or platelet concentration (%) = PRP Platelet count/ Whole blood Platelet count of the given sample x 100.

The SPSS version 23 was used to enter and analyze the data. The mean and standard deviation for platelet, RBCs and WBCs counts and platelet yield was determined using Independent Samples T-Test and p value <0.01 was taken as significant.

## RESULTS

Among 50 participants, the mean age ± 2SD was 28.58 ± 11.78 years with male to female ratio of 1.7:1. The mean whole blood platelet count was 345.6 ± 132.6 ×10^3^/μl. The mean PRP platelet count in Group-I was 594.6 ± 157.4×10^3^/μl whereas in Group-II was 923.06 ± 127.58×10^3^/μl. The mean platelet concentration/yield in Group-I was 175.75 ± 55.08% and in the Group-II was 276.78±112.7%. The significant differences were observed between the platelet counts and platelet yields from the PRP samples of Group-s I and II (p < 0.01) (Table-I).

**Table-I:** Comparison between mean and standard deviation of Platelet count and platelet concentration/ yield from Group-I and II.

Parameters	Single spin-Group-I (Mean ±2SD)	Double spin-Group-II (Mean ±2SD)	p-value
Platelet Count	594.6±157.4 x10^3^/µl	923.06±127.58 x10^3^/µl	<0.01
Platelet Yield	175.75 ± 55.08%	276.78±112.7%	<0.01

WBCs count was higher in PRP prepared by single spin method (Group-I). Significant difference between the WBCs counts from the PRP samples of Group-s I and II (p < 0.01) was observed. However, there was insignificant difference in the RBCs count of Group-I and Group-II PRP samples (p=0.139) (Table-II).

**Table-II:** Comparison between standard deviation and mean of WBCs and RBCs from Group-I and II.

Parameters	Single spin-Group-I (Mean ±SD)	Double spin-Group-II (Mean ±SD)	p-value
WBCs	6.0560±8.45713 x10^3^/µl	1.0600± 0.31403 x10^3^/µl	< 0.01
RBCs	0.0588 ± 0.06882 x10^6^/µl	0.0436± 0.02447 x10^6^/µl	0.139

## DISCUSSION

The effective PRP term comes under the umbrella of “Therapeutic PRP” which is defined as having one million platelets per microliter. In a healthy individual, the platelet count ranges from 150-400 x10^3^/μl. So, a well prepared PRP contains about five times higher platelets which are much more enriched with growth factors than venous blood.^12^ In previous studies, different protocols for preparation of PRP are explained in terms of centrifugal force and time. These different approaches have resulted in platelet concentrates of varying platelet counts. For standardizing the regenerative capacity of PRP, the platelet count should be one of the main factors.

The platelets damaged during processing will not secrete bioactive growth factors leading to inadequate outcome.^13^ The present study evaluated the quantity of platelets in PRP samples prepared according to two different protocols (single spin and double spin). In the present study, the mean PRP platelet count in Group-I was 594.6 ± 157.4×10^3^/μl and in Group-II was 923.06 ± 127.58×10^3^/μl with significant difference seen among two Group-s in this regard (p < 0.01). Higher trend was found in Group-II. In this study, only the double centrifugation method used in Group-II produced a “therapeutic PRP”.

Our findings are similar to Nagata MJ et al., who have reported mean platelet count in PRP prepared by double centrifugation as 1,986.87 ± 685 x 10^3^/μl, much higher than other Group of single-spin PRP. The higher count reported may be the reflection of higher whole blood platelet count, which they reported as 454.68 ± 181.538x10^3^/μl while in our study it was found as 345.6 ± 132.6 ×10^3^/μl.^11^ Marx et al. have recommended double-centrifugation to obtain concentrated platelets from whole blood.^14^ However, optimal therapeutic effects of PRP in bone regeneration prepared by single-spin technique have been documented but the platelet counts are not mentioned.^8^

The commercially available PRP production systems have been investigated for optimization. These systems are less laborious but costly.^15^

The platelet counts obtained are similar to that prepared by simple tube spin method so enhancing the fact that these simple methods can be adopted in the resource constrained settings. We found the mean platelet concentration/yield in Group-I and II as 175.75 ± 55.08% and 276.78 ± 112.7% respectively, showing significantly higher trend for Group-II samples. This parameter depicted the percentage of concentration or increase of platelet count in the PRP sample in reference to the respective whole blood sample. The measure was based on the study by Tamimi et al and Nagata MJ et al.^10^,^11^ Harrison et al. exclusively reviewed only the different single-spin techniques and reported PRP platelet yield range of 50%-72%. They also showed relation of lower whole blood hematocrit with higher platelet yield.^8^

Other study has shown higher percentage of platelet concentration for samples prepared from double centrifugation method concordant with our results.^9^ The results of our study revealed that the residual WBCs count was higher in PRP prepared by single spin method with significant difference between Group-s I and II (p < 0.01). Previous study has demonstrated presence of activated lymphocytes in smears of PRP of both Group-s.^11^ Higher neutrophils can aid in healing chronic inflammatory disorders.^16^ Other studies found the PRP with high neutrophils count, known as leukocyte (neutrophil)-rich PRP (LR-PRP), can lead to pro-inflammatory state.^17^

The raised neutrophils in LR-PRP can also cause elevation of certain catastrophic cytokines, including interleukin-1β, tumor necrosis factor-α, and metalloproteinases, which may upset the regenerative effect of platelets.^18^ Less contamination of double-centrifuged PRP by neutrophils and lymphocytes provides benefit in terms of less chance of inflammatory cytokines initiation. Although so far, previous studies have focused on autologous PRP source, but in cases where PRP is required from another donor, minimal WBCs is an important factor to prevent alloimmunization.

### Limitation:

The limitations of the study included time- bound sample collection, so the therapeutic effects of two Group-s of PRP could not be determined. The platelet function studies can also provide a valuable information. Double-centrifugation method has proven to be better technique but a standardized centrifugal force and centrifugation time determination needs more research in this direction from multiple centers.

## CONCLUSION

The effect of Platelet rich plasma (PRP) on wound healing depends on many variables including the protocol used for its preparation. The study concluded that the double centrifugation method resulted in higher platelet quantity and yield with less contamination by red and white blood cells than did the single centrifugation method for PRP preparation. So, double centrifugation method is beneficial in preparation of autologous as well as allogenic PRP on the basis of lower counts of RBCs and WBCs.

### Authors Contribution:

**NS:** Designed & Contributed in manuscript writing. She is also responsible for the integrity and accuracy of the study.

**NM:** Review of manuscript and literature search.

**TF:** Collection & assembly of data.

**AS:** Data Collection, Manuscript writing.
